# Dietary arginine-to-lysine ratio influences nutrient digestibility during early growth without affecting growth performance in growing–finishing pigs

**DOI:** 10.3389/fvets.2026.1973474

**Published:** 2026-09-25

**Authors:** Wei Han Zhao, Joo Hee Kim, In Ho Kim

**Affiliations:** 1 Department of Animal Biotechnology, Dankook University, Cheonan, Republic of Korea; 2 Smart Animal Bio Institute, Dankook University, Cheonan, Republic of Korea

**Keywords:** arginine, creatine, growing–finishing pigs, homocysteine, lysine, nutrient digestibility

## Abstract

The balance of dietary amino acid ratios is an important factor affecting nutrient utilization in pigs. Both arginine and lysine are involved in protein deposition and nitrogen metabolism, and their relative ratio may influence nutrient utilization and metabolic responses. This study evaluated the effects of different dietary arginine-to-lysine ratios (Arg:Lys) on growth performance, fecal characteristics, nutrient digestibility, and serum metabolites in growing–finishing pigs. A total of 165 pigs [Duroc × (Landrace × Yorkshire)] with an initial body weight (BW) of 26.36 ± 3.01 kg were assigned to three dietary treatments in a randomized complete block design based on BW. The CON, TRT1, and TRT2 diets were formulated to provide Arg:Lys ratios of 1.00, 1.10, and 1.20, respectively. Each treatment had 11 replicate pens, with 5 pigs per pen, including 2 gilts and 3 barrows. The experiment lasted 15 weeks. Dietary Arg:Lys ratio did not affect BW, average daily gain, average daily feed intake, or feed conversion ratio during any experimental period (*p* > 0.05). Fecal score was not affected by dietary treatment throughout the experiment (*p* > 0.05). Pigs fed the TRT1 diet had greater apparent total tract digestibility (ATTD) of nitrogen (N) (*p* = 0.0247, *p* = 0.0361) and gross energy (GE) (*p* = 0.0402, *p* = 0.0343) at weeks 2 and 5 than pigs fed the CON diet. At week 12, ATTD of dry matter (DM) showed a significant quadratic response (*p* = 0.0371). Fecal moisture was not affected at weeks 2, 5, 9, or 12, but showed a significant quadratic response at week 15 (*p* = 0.0230). Serum homocysteine concentration showed a significant quadratic response at week 5 (*p* = 0.0334), whereas serum creatine concentration was not affected. Overall, dietary Arg:Lys ratio did not affect growth performance in growing–finishing pigs, but an Arg:Lys ratio of 1.10 improved the ATTD of N and GE during the early growing phase. However, because these digestibility responses were not maintained throughout the entire experimental period and were not accompanied by improvements in growth performance, their practical nutritional significance requires further investigation.

## Introduction

Dietary amino acid balance is an important factor affecting growth performance, protein deposition, and nutrient utilization in pigs (1). Compared with the absolute supply of a single amino acid, the relative proportions among amino acids may more accurately reflect the adequacy of dietary protein nutrition (2). In swine diet formulation, lysine is commonly used as the reference amino acid because it is often the first limiting amino acid in corn–soybean meal-based diets and is closely associated with lean tissue deposition (3). Therefore, establishing appropriate amino acid ratios relative to lysine may improve amino acid utilization and reduce nutrient wastage and nitrogen excretion (4). However, the appropriate ratios between functional amino acids and lysine may vary depending on growth stage, diet composition, and metabolic demand (5).

Arginine is involved not only in protein synthesis but also in metabolic pathways related to creatine synthesis, the urea cycle, muscle energy metabolism, and nitrogen metabolism (6). Therefore, dietary arginine supply may affect nutrient utilization and metabolic status in pigs. Because arginine and lysine are both cationic amino acids and may share some transport systems, their relative ratio may influence amino acid balance (7). Thus, maintaining an appropriate dietary Arg:Lys ratio may be important for supporting the nutritional functions of arginine. Previous studies have shown that dietary arginine supplementation can affect growth performance, intestinal development, immune function, and metabolic responses in pigs, particularly during weaning and other stress-related conditions (8). However, most previous studies have focused on the absolute amount of arginine supplementation rather than its proportion relative to lysine. Because lysine is commonly used as the reference amino acid in swine diet formulation, evaluating the Arg:Lys ratio may provide a more balanced approach to assessing arginine supply within the overall amino acid profile. Information on the responses of growing–finishing pigs to different dietary Arg:Lys ratios under practical feeding conditions remains limited.

Therefore, this study was conducted to evaluate the effects of different dietary Arg:Lys ratios on growth performance, fecal score, fecal moisture, nutrient digestibility, and serum metabolites in growing–finishing pigs, and to determine whether changes in nutrient digestibility were accompanied by corresponding changes in growth performance. We hypothesized that a moderate increase in dietary Arg:Lys ratio would influence nutrient digestibility and related metabolic responses without adversely affecting growth performance or fecal characteristics.

## Materials and methods

### Ethics approval

The experiment was conducted at the Dankook University experimental farm in Jeonui, Sejong, Republic of Korea. All animal procedures were reviewed and approved by the Institutional Animal Care and Use Committee of Dankook University (Approval No. DK-1-2516_1, 2; approved on August 15, 2025).

### Source of amino acids

The crystalline amino acids used in the diets were supplied by Jeil Feed Co., Ltd. (Daejeon, Republic of Korea).

### Study design

A total of 165 pigs [Duroc × (Landrace × Yorkshire)] with an initial BW of 26.36 ± 3.01 kg were used in a randomized complete block design. Pigs were blocked based on initial BW and randomly assigned to three dietary treatments: CON (Arg:Lys = 1.00), TRT1 (Arg:Lys = 1.10), and TRT2 (Arg:Lys = 1.20). Each treatment had 11 replicate pens, with 5 pigs per pen, including 2 gilts and 3 barrows. The trial lasted for 15 weeks. All diets were formulated to meet or exceed the nutrient requirements for growing–finishing pigs recommended by the National Research Council (9), and their ingredient and nutrient compositions are presented in Tables 1–3.

**Table 1:** Composition of experimental diets for growing pigs.

Item	Growing pig		
	CON (Arg:Lys = 1.00)	TRT1 (Arg:Lys = 1.10)	TRT2 (Arg:Lys = 1.20)
Ingredients (%)			
Corn	70.08	70.64	71.18
Soybean meal	15.37	14.65	13.92
DDGS	4.00	4.00	4.00
Palm kernel meal	4.00	4.00	4.00
Tallow	0.99	0.96	0.95
Molasses	2.00	2.00	2.00
MDCP	1.01	1.02	1.04
Limestone	0.86	0.86	0.86
Lysine (78%)	0.55	0.57	0.60
Methionine (98%)	0.06	0.06	0.06
Threonine (98%)	0.17	0.19	0.20
Tryptophan (98%)	0.03	0.04	0.04
Arginine (98.5%)	0.18	0.31	0.45
Salt	0.20	0.20	0.20
Mineral mix^1^	0.20	0.20	0.20
Vitamin mix^2^	0.20	0.20	0.20
Choline (25%)	0.10	0.10	0.10
Total	100.00	100.00	100.00
Calculated value			
Crude protein, %	16.00	16.00	16.00
Metabolizable energy, kcal/kg	3,300	3,300	3,300
Calcium, %	0.66	0.66	0.66
Phosphorus, %	0.56	0.56	0.56
Lysine, %	1.12	1.12	1.12
Methionine, %	0.32	0.32	0.32
Threonine, %	0.72	0.72	0.72
Tryptophan, %	0.19	0.19	0.19
Arginine, %	1.12	1.23	1.34
Crude fat, %	4.13	4.11	4.10
Crude fiber, %	3.09	3.07	3.06
Crude ash, %	4.14	4.11	4.09

1 Provided per kg diet: Fe, 100 mg as ferrous sulfate; Cu, 17 mg as copper sulfate; Mn, 17 mg as manganese oxide; I, 0.5 mg as potassium iodide; and Se, 0.3 mg as sodium selenite.

2 Provided per kilograms of diet: vitamin A, 10,800 IU; vitamin D_3_, 4,000 IU; vitamin E, 40 IU; vitamin K_3_, 4 mg; vitamin B1, 6 mg; vitamin B2, 12 mg; vitamin B_6_, 6 mg; vitamin B_12_, 0.05 mg; biotin, 0.2 mg; folic acid, 2 mg; niacin, 50 mg; D-calcium pantothenate, 25 mg.

**Table 2:** Composition of experimental diets for finishing pigs.

Item	Finishing pig (phase 1)		
	CON (Arg:Lys = 1.00)	TRT1 (Arg:Lys = 1.10)	TRT2 (Arg:Lys = 1.20)
Ingredients (%)			
Corn	72.57	73.09	73.53
Soybean meal	13.64	12.97	12.39
DDGS	4.00	4.00	4.00
Palm kernel meal	4.00	4.00	4.00
Tallow	0.81	0.77	0.76
Molasses	2.00	2.00	2.00
MDCP	0.85	0.86	0.87
Limestone	0.78	0.78	0.78
Lysine (78%)	0.41	0.44	0.46
Methionine (98%)	0.02	0.03	0.03
Threonine (98%)	0.12	0.13	0.14
Tryptophan (98%)	0.02	0.03	0.03
Arginine (98.5%)	0.08	0.20	0.31
Salt	0.20	0.20	0.20
Mineral mix^1^	0.20	0.20	0.20
Vitamin mix^2^	0.20	0.20	0.20
Choline (25%)	0.10	0.10	0.10
Total	100.00	100.00	100.00
Calculated value			
Crude protein, %	15.00	15.00	15.00
Metabolizable energy, kcal/kg	3,300	3,300	3,300
Calcium, %	0.52	0.52	0.52
Phosphorus, %	0.47	0.47	0.47
Lysine, %	0.97	0.97	0.97
Methionine, %	0.28	0.28	0.28
Threonine, %	0.64	0.64	0.64
Tryptophan, %	0.17	0.17	0.17
Arginine, %	0.97	1.07	1.16
Crude fat, %	4.01	3.98	3.97
Crude fiber, %	3.07	3.06	3.04
Crude ash, %	3.85	3.83	3.80

1 Provided per kg diet: Fe, 100 mg as ferrous sulfate; Cu, 17 mg as copper sulfate; Mn, 17 mg as manganese oxide; I, 0.5 mg as potassium iodide; and Se, 0.3 mg as sodium selenite.

2 Provided per kilograms of diet: vitamin A, 10,800 IU; vitamin D_3_, 4,000 IU; vitamin E, 40 IU; vitamin K_3_, 4 mg; vitamin B1, 6 mg; vitamin B2, 12 mg; vitamin B_6_, 6 mg; vitamin B_12_, 0.05 mg; biotin, 0.2 mg; folic acid, 2 mg; niacin, 50 mg; D-calcium pantothenate, 25 mg.

**Table 3:** Composition of experimental diets for finishing pigs.

Item	Finishing pig (phase 2)		
	CON (Arg:Lys = 1.00)	TRT1 (Arg:Lys = 1.10)	TRT2 (Arg:Lys = 1.20)
Ingredients (%)			
Corn	78.41	78.80	79.21
Soybean meal	8.25	7.72	7.16
DDGS	4.00	4.00	4.00
Palm kernel meal	4.00	4.00	4.00
Tallow	0.50	0.49	0.49
Molasses	2.00	2.00	2.00
MDCP	0.71	0.73	0.74
Limestone	0.71	0.71	0.71
Lysine (78%)	0.43	0.45	0.47
Methionine (98%)	0.02	0.02	0.02
Threonine (98%)	0.12	0.13	0.14
Tryptophan (98%)	0.04	0.04	0.04
Arginine (98.5%)	0.11	0.21	0.32
Salt	0.20	0.20	0.20
Mineral mix^1^	0.20	0.20	0.20
Vitamin mix^2^	0.20	0.20	0.20
Choline (25%)	0.10	0.10	0.10
Total	100.00	100.00	100.00
Calculated value			
Crude protein, %	13.00	13.00	13.00
Metabolizable energy, kcal/kg	3,300	3,300	3,300
Calcium, %	0.52	0.52	0.52
Phosphorus, %	0.47	0.47	0.47
Lysine, %	0.84	0.84	0.84
Methionine, %	0.25	0.25	0.25
Threonine, %	0.56	0.56	0.56
Tryptophan, %	0.15	0.15	0.15
Arginine, %	0.84	0.92	1.01
Crude fat, %	3.82	3.82	3.83
Crude fiber, %	2.98	2.97	2.95
Crude ash, %	3.41	3.40	3.38

1 Provided per kg diet: Fe, 100 mg as ferrous sulfate; Cu, 17 mg as copper sulfate; Mn, 17 mg as manganese oxide; I, 0.5 mg as potassium iodide; and Se, 0.3 mg as sodium selenite.

2 Provided per kilograms of diet: vitamin A, 10,800 IU; vitamin D_3_, 4,000 IU; vitamin E, 40 IU; vitamin K_3_, 4 mg; vitamin B1, 6 mg; vitamin B2, 12 mg; vitamin B_6_, 6 mg; vitamin B_12_, 0.05 mg; biotin, 0.2 mg; folic acid, 2 mg; niacin, 50 mg; D-calcium pantothenate, 25 mg.

All pigs were housed in a temperature-controlled facility equipped with plastic slatted floors and a mechanical ventilation system. The ventilation system was regulated by an automatic airflow control system to ensure adequate air exchange and reduce the accumulation of harmful gasses. Each pen measured 3.2 × 2.0 m and was equipped with a stainless-steel self-feeder and a nipple drinker to allow ad libitum access to feed and water. Throughout the 15-week experimental period, relative humidity was maintained at 60 to 70%. Artificial lighting was provided for 12 h per day, with lights on at 09:00 h and off at 21:00 h, and the ambient temperature was maintained at 23 °C. Routine inspections were conducted two times daily to ensure adequate feed and water supply and to monitor pig health.

### Growth performance

BW of each pig was measured using a portable electronic scale (GL-6000, CAS Co., Ltd., Seoul, Republic of Korea) at the beginning of the experiment and at weeks 2, 5, 6, 9, 12, and 15. These values were used to calculate ADG for each experimental period and for the overall period. Feed intake and feed refusals were recorded by pen to determine ADFI, which was subsequently used to calculate FCR.

$$\mathrm{ADG}\;(g/d)=\frac{\text{Final}\;\mathrm{BW}-\text{Initial}\;\mathrm{BW}\;(g)}{\text{Experimental days}\;}$$

$$\text{ADFI}(g/d)=\frac{\text{Total Feed Intake}\;(g)}{\text{Experimental days}\;}$$

$$\mathrm{FCR}=\frac{\text{Feed Intake}\;}{\text{Weight Gain}\;}$$

### Fecal score

During weeks 1 to 15, fecal scores were recorded for 7 consecutive days each week according to the method described by Hu et al. (10). At the same time each morning, 2 pigs were randomly selected from each pen and independently scored by 2 trained evaluators. The scores assigned by the 2 evaluators were recorded separately and averaged for each pig, and the mean score of the 2 pigs was then used as the daily fecal score for that pen. The daily pen scores recorded over 7 consecutive days were then averaged to obtain the weekly fecal score for each pen. Fecal score was assessed using a 5-point scale as follows: 1 = hard, dry, pellet-like feces; 2 = firm and well-formed feces; 3 = soft, moist feces that retained their shape; 4 = loose, unformed feces; and 5 = watery or liquid feces. The pen was considered the experimental unit for fecal score.

### Nutrient digestibility

To determine ATTD, measurements were conducted at weeks 2, 5, 9, 12, and 15. For each sampling period, 0.50% chromium oxide (Cr₂O₃) was included in the diets for 7 consecutive days, with the first 4 d used for marker adaptation and fecal samples collected during the final 3 d. Feed samples were collected in sterile bags and stored until analysis. Fecal samples were collected by rectal massage at the same time each morning from 1 gilt and 1 barrow in each replicate pen for 3 consecutive days, and samples were pooled by pen. After collection, fecal samples were immediately placed in sealed sampling bags and transferred to the laboratory.

For fecal moisture determination, fresh fecal samples were thoroughly mixed immediately after collection. An appropriate amount of each sample was weighed to record the fresh weight. Samples were then dried in a forced-air drying oven at 105 °C for 24 h to constant weight, and the dry weight was recorded. Fecal moisture was calculated using the following equation:

$$\text{Fecal moisture}\;(\%)=\frac{\text{Fresh weight}-\mathrm{Dry}\;\text{weight}}{\text{Fresh weight}}\times 100$$

For nutrient digestibility analysis, fecal samples pooled by replicate pen were stored at −20 °C until analysis of DM, N, GE, and chromium concentrations. Before chemical analysis, all samples were dried at 60 °C for 72 h for preservation and further processing, ground using a Wiley mill, and passed through a 1-mm screen to ensure sample homogeneity. To determine DM content, subsamples were dried in a forced-air drying oven at 105 °C for 24 h. The DM, N, and GE contents of feed and fecal samples were analyzed according to the standard methods of AOAC (11). Nitrogen was determined using the Kjeldahl method with a Tecator™ Kjeltec 8,400 analyzer (Hoeganaes, Sweden), and GE was determined using a Parr 6,400 bomb calorimeter (Parr Instrument Co., Moline, IL, USA). Chromium concentration was measured using a UV–visible spectrophotometer (UV-1201; Shimadzu Corp., Kyoto, Japan). The ATTD of each nutrient was calculated using the indicator method.

$$\text{ATTD}\;(\%)=[1-\{(N_{f}\times C_{d})/(N_{d}\times C_{f})\}]\times 100$$

where N_f_ represents nutrient concentration in feces (% DM), C_d_ is chromium concentration in the diet (% DM), N_d_ is nutrient concentration in the diet (% DM), and *C_f_* is chromium concentration in feces (% DM).

### Serum metabolites

Blood samples were collected at weeks 2, 5, and 15. At week 2, 1 pig was randomly selected from each replicate pen, and the same pig was sampled again at weeks 5 and 15. Blood samples were collected from the jugular vein by trained personnel while pigs were restrained in a standing position. All pigs were sampled under nonfasted conditions, and blood collection from all treatments was completed within the same time window on each sampling day. Before sampling, the puncture site was disinfected with 70% ethanol, and blood was collected using a 10-mL sterile disposable syringe (HLB Life Science Co., Ltd., Hwaseong, Republic of Korea). A 5-mL aliquot of blood was transferred into a vacuum tube without anticoagulant (Becton Dickinson Vacutainer Systems, Franklin Lakes, NJ, USA). Blood samples were allowed to clot at room temperature for 30 min and then centrifuged at 4,000 × g for 15 min at 4 °C. The separated serum was transferred into sterile centrifuge tubes and stored at −20 °C until analysis.

Serum creatine and homocysteine concentrations were determined using commercial assay kits (Shanghai Yuanju Biotechnology Center, Shanghai, China) according to the manufacturer’s instructions. All samples were analyzed under the same laboratory conditions.

### Statistical analysis

All data were analyzed using SAS software (version 9.4; SAS Institute Inc., Cary, NC, USA). Before ANOVA, for each response variable, the normality of model residuals was assessed using the Shapiro–Wilk test, and homogeneity of variance was evaluated using Levene’s test. For growth performance, fecal score, fecal moisture, and nutrient digestibility, the pen was considered the experimental unit. The statistical model included dietary treatment as the fixed effect and initial BW block as the random effect. Data collected at different measurement times or experimental periods were analyzed separately. For serum metabolites, 1 pig sampled from each pen was used to represent the pen, and serum metabolite data at weeks 2, 5, and 15 were analyzed separately. Orthogonal polynomial contrasts were used to evaluate the linear and quadratic effects of increasing dietary Arg:Lys ratio. When significant treatment effects were detected by ANOVA, treatment means were compared using Tukey’s test. Statistical significance was declared at *p* < 0.05.

## Results

### Growth performance

The effects of dietary Arg:Lys ratios on growth performance in growing–finishing pigs are shown in Table 4. Initial BW did not differ among treatments. Dietary Arg:Lys ratio had no significant effect on BW throughout the 15-week experimental period. Similarly, ADG, ADFI, and FCR were not affected by dietary Arg:Lys ratio during the overall experimental period.

**Table 4:** Effects of dietary Arg:Lys ratio on growth performance in growing–finishing pigs^1^.

Items	CON	TRT1	TRT2	SEM	*p*-value	
					Linear	Quadratic
Body weight, kg						
Initial	26.37	26.36	26.35	0.04	0.7205	0.9039
Week 2	36.23	36.29	36.52	0.26	0.4427	0.8158
Week 5	51.52	52.30	52.12	0.74	0.5731	0.5955
Week 6	56.91	57.83	57.54	0.91	0.6320	0.5896
Week 9	73.70	75.13	74.72	1.58	0.6543	0.6398
Week 12	91.76	92.86	93.11	2.32	0.6851	0.8820
Week 15	110.34	112.12	112.52	3.09	0.6232	0.8593
Initial – Week 2						
ADG, g/d	704	709	726	19	0.4149	0.7928
ADFI, g/d	1,312	1,281	1,293	28	0.6373	0.5291
FCR	1.866	1.813	1.784	0.030	0.0646	0.7475
Week 2 – Week 5						
ADG, g/d	728	762	743	24	0.6657	0.3654
ADFI, g/d	1,619	1,648	1,631	33	0.8060	0.5725
FCR	2.232	2.171	2.208	0.032	0.5974	0.2154
Week 5 – Week 6						
ADG, g/d	769	785	774	28	0.8989	0.6991
ADFI, g/d	1,713	1,728	1,710	44	0.9535	0.7684
FCR	2.248	2.220	2.226	0.043	0.7248	0.7543
Week 6 – Week 9						
ADG, g/d	800	824	817	33	0.7177	0.7042
ADFI, g/d	1,976	2,011	1,983	55	0.9360	0.6412
FCR	2.491	2.461	2.443	0.044	0.4566	0.9140
Week 9 – Week 12						
ADG, g/d	859	844	874	37	0.7722	0.6127
ADFI, g/d	2,392	2,416	2,405	66	0.8930	0.8263
FCR	2.807	2.900	2.778	0.057	0.7231	0.1434
Week 12 – Week 15						
ADG, g/d	885	917	925	40	0.4932	0.8083
ADFI, g/d	2,764	2,780	2,817	72	0.6104	0.9077
FCR	3.160	3.065	3.078	0.062	0.3609	0.4892
Overall						
ADG, g/d	800	817	821	30	0.6270	0.8613
ADFI, g/d	2,039	2,057	2,053	48	0.8390	0.8582
FCR	2.565	2.536	2.518	0.036	0.3644	0.8968

1 CON, Arg:Lys ratio = 1.00; TRT1, Arg:Lys ratio = 1.10; TRT2, Arg:Lys ratio = 1.20; SEM, standard error of the mean.

### Fecal scores

The effects of dietary Arg:Lys ratios on fecal score in growing–finishing pigs are shown in Table 5. Dietary Arg:Lys ratio did not significantly affect fecal score from weeks 1 to 15.

**Table 5:** Effects of dietary Arg:Lys ratio on fecal score in growing–finishing pigs^1^.

Items	CON	TRT1	TRT2	SEM	*p*-value	
					Linear	Quadratic
Fecal score^2^						
Week 1	3.35	3.37	3.35	0.02	1.0000	0.4829
Week 2	3.32	3.33	3.34	0.03	0.6100	0.9216
Week 3	3.29	3.27	3.28	0.03	0.7396	0.6811
Week 4	3.27	3.27	3.24	0.02	0.2891	0.5151
Week 5	3.25	3.25	3.26	0.03	0.8257	0.8988
Week 6	3.23	3.26	3.22	0.02	0.8382	0.2464
Week 7	3.19	3.22	3.18	0.02	0.8644	0.2324
Week 8	3.15	3.18	3.19	0.02	0.2600	0.6788
Week 9	3.13	3.16	3.17	0.03	0.3165	0.8595
Week 10	3.13	3.14	3.15	0.03	0.5847	0.9083
Week 11	3.10	3.12	3.09	0.01	0.7567	0.2188
Week 12	3.09	3.12	3.11	0.02	0.5285	0.5440
Week 13	3.07	3.10	3.08	0.02	0.6615	0.3004
Week 14	3.08	3.11	3.06	0.02	0.5334	0.1285
Week 15	3.09	3.09	3.08	0.01	0.4822	0.6837

1 CON, Arg:Lys ratio = 1.00; TRT1, Arg:Lys ratio = 1.10; TRT2, Arg:Lys ratio = 1.20; SEM, standard error of the mean.

2 Fecal score = 1 hard, dry pellet; 2 firm, formed stool; 3 soft, moist stool that retains shape; 4 soft, unformed stool that assumes shape of container; 5 watery liquid that can be poured.

### Nutrient digestibility

The effects of dietary Arg:Lys ratios on nutrient digestibility in growing–finishing pigs are shown in Table 6. At week 2, pigs fed the TRT1 diet had greater ATTD of N than those fed the CON diet, whereas TRT2 showed intermediate values; a significant quadratic effect was observed for ATTD of N (*p* = 0.0247). Similarly, ATTD of GE at week 2 was greater in TRT1 than in CON, with TRT2 showing intermediate values, and a significant quadratic effect was observed (*p* = 0.0402). At week 5, dietary Arg:Lys ratio had no significant effect on ATTD of DM. However, compared with CON, TRT1 increased ATTD of N, whereas TRT2 showed intermediate values; this response was associated with a significant quadratic effect (*p* = 0.0361). A similar pattern was observed for ATTD of GE, with greater values in TRT1 than in CON and intermediate values in TRT2, accompanied by a significant quadratic effect (*p* = 0.0343). At week 9, dietary Arg:Lys ratio did not significantly affect ATTD of DM, N, or GE. At week 12, ATTD of DM showed a significant quadratic response (*p* = 0.0371), with the greatest numerical value observed in TRT1, whereas ATTD of N and GE were not significantly affected. At week 15, no significant differences in ATTD of DM, N, or GE were observed among treatments.

**Table 6:** Effects of dietary Arg:Lys ratio on nutrient digestibility in growing–finishing pigs^1^.

Items, %	CON	TRT1	TRT2	SEM	*p*-value	
					Linear	Quadratic
Week 2						
Dry matter	70.64	73.98	73.69	1.11	0.0811	0.5426
Nitrogen	65.58^b^	70.40^a^	67.22^ab^	1.32	0.3887	0.0247
Gross energy	67.03^b^	71.53^a^	69.27^ab^	1.28	0.2156	0.0402
Week 5						
Dry matter	68.38	70.73	69.57	1.05	0.3623	0.1281
Nitrogen	64.98^b^	67.57^a^	65.53^ab^	1.32	0.7151	0.0361
Gross energy	65.52^b^	68.54^a^	66.95^ab^	1.18	0.2927	0.0343
Week 9						
Dry matter	64.92	68.57	66.13	1.28	0.5197	0.0813
Nitrogen	61.74	65.70	63.86	1.45	0.3238	0.1316
Gross energy	62.43	66.20	63.88	1.39	0.4768	0.1039
Week 12						
Dry matter	62.63	67.45	64.47	1.34	0.3485	0.0371
Nitrogen	59.01	64.21	62.87	1.50	0.0931	0.1004
Gross energy	59.47	65.35	63.68	1.77	0.1157	0.1040
Week 15						
Dry matter	61.08	65.59	63.09	1.60	0.3956	0.1048
Nitrogen	56.36	60.16	61.14	1.78	0.0870	0.5322
Gross energy	56.93	59.73	61.38	1.70	0.0932	0.7905

1 CON, Arg:Lys ratio = 1.00; TRT1, Arg:Lys ratio = 1.10; TRT2, Arg:Lys ratio = 1.20; SEM, standard error of the mean.

^a,b^ Means in the same row with different superscripts differ significantly (*p* < 0.05).

### Fecal moisture

The effects of dietary Arg:Lys ratios on fecal moisture in growing–finishing pigs are shown in Table 7. Dietary Arg:Lys ratio did not significantly affect fecal moisture at weeks 2, 5, 9, or 12. At week 15, fecal moisture showed a significant quadratic response (*p* = 0.0230), with the greatest numerical value observed in TRT1 and the lowest numerical value observed in TRT2.

**Table 7:** Effects of dietary Arg:Lys ratio on fecal moisture in growing–finishing pigs^1^.

Items	CON	TRT1	TRT2	SEM	*p*-value	
					Linear	Quadratic
Fecal moisture, %						
Week 2	53.82	52.60	50.65	2.02	0.3090	0.8868
Week 5	49.07	44.29	48.46	2.16	0.8493	0.1409
Week 9	64.80	64.29	63.90	0.36	0.1274	0.8986
Week 12	62.85	61.43	63.37	0.74	0.6353	0.1126
Week 15	60.02^b^	61.88^a^	58.99^ab^	0.64	0.2949	0.0230

1 CON, Arg:Lys ratio = 1.00; TRT1, Arg:Lys ratio = 1.10; TRT2, Arg:Lys ratio = 1.20; SEM, standard error of the mean.

^a,b^ Means in the same row with different superscripts differ significantly (*p* < 0.05).

### Serum metabolites

The effects of dietary Arg:Lys ratios on serum metabolites in growing–finishing pigs are shown in Table 8. At week 2, serum creatine and homocysteine concentrations did not differ among treatments. At week 5, serum creatine concentration was not affected by dietary Arg:Lys ratio. However, serum homocysteine concentration showed a significant quadratic response (*p* = 0.0334), with a greater value in TRT1 than in CON and an intermediate value in TRT2. At week 15, dietary Arg:Lys ratio had no significant effect on serum creatine or homocysteine concentrations.

**Table 8:** Effects of dietary Arg:Lys ratio on serum metabolites in growing–finishing pigs^1^.

Items	CON	TRT1	TRT2	SEM	*p*-value	
					Linear	Quadratic
Week 2						
Creatine, mg/dL	0.80	0.85	0.80	0.04	1.0000	0.4170
Homocysteine, μmol/L	15.10	18.88	17.32	2.10	0.4760	0.3294
Week 5						
Creatine, mg/dL	1.15	1.16	1.18	0.08	0.7639	0.8987
Homocysteine, μmol/L	13.14^b^	16.22^a^	13.44^ab^	1.12	0.8350	0.0334
Week 15						
Creatine, mg/dL	1.56	1.49	1.61	0.08	0.6806	0.3858
Homocysteine, μmol/L	23.42	20.34	20.24	1.36	0.1375	0.3980

1 CON, Arg:Lys ratio = 1.00; TRT1, Arg:Lys ratio = 1.10; TRT2, Arg:Lys ratio = 1.20; SEM, standard error of the mean.

^a,b^Means in the same row with different superscripts differ significantly (*p* < 0.05).

## Discussion

Arginine is involved in protein synthesis and N metabolism, and its nutritional effects may be influenced by basal dietary amino acid supply, growth stage, and physiological status (12). In the present study, increasing the dietary Arg:Lys ratio from 1.00 to 1.10 or 1.20 did not significantly affect BW, ADG, ADFI, or FCR in growing–finishing pigs throughout the 15-week experimental period. Similarly, previous studies have reported that supplementation with L-arginine did not significantly improve growth performance in pigs (13), which is consistent with the present results. In contrast, other studies have shown that increasing arginine supply can improve growth performance in piglets under conditions of greater physiological demand, such as early weaning or heat stress (14, 15). These inconsistent findings suggest that the growth response to arginine may depend on animal age, health status, environmental conditions, and basal dietary arginine supply. In the present study, the three diets had the same calculated crude protein, metabolizable energy, and lysine levels and were formulated to meet the nutrient requirements of growing–finishing pigs. Therefore, the arginine supply in the basal diet may have been sufficient to support normal growth under the present experimental conditions, and further increasing the Arg:Lys ratio did not provide additional growth benefits.

Regarding nutrient digestibility, pigs fed the diet with an Arg:Lys ratio of 1.10 had greater ATTD of N and GE at weeks 2 and 5 than those fed the CON diet, whereas further increasing the ratio to 1.20 did not provide additional improvement. Previous studies have reported inconsistent effects of arginine supplementation on nutrient utilization, with some studies observing improved intestinal digestive and absorptive function (16, 17), whereas others found no significant effects on nutrient digestibility (15). These differences may be related to diet composition, arginine supply level, growth stage, and basal diet digestibility. In the present study, the improvement in ATTD of N and GE was mainly observed during the early growing phase and was not consistently maintained during the later finishing phase. At week 12, ATTD of DM showed a quadratic response, but no consistent improvement in ATTD of N or GE was observed at weeks 9, 12, or 15. These results suggest that the digestibility response to dietary Arg:Lys ratio may be stage-dependent. As pigs grow from the growing to finishing phase, changes in digestive capacity, potential changes in Arg:Lys requirements, maintenance requirements, and nutrient partitioning may reduce the detectable effect of Arg:Lys ratio on nutrient digestibility.

Notably, the early improvement in ATTD of N and GE was not accompanied by improved growth performance. This may be because ATTD and growth performance reflect different physiological processes. ATTD represents the apparent disappearance of nutrients across the total digestive tract, whereas growth performance is the integrated outcome of feed intake, digestion, absorption, maintenance metabolism, and nutrient deposition in the body (18). Therefore, greater ATTD of N does not necessarily indicate a parallel improvement in N retention or protein synthesis efficiency (19, 20), and greater ATTD of GE cannot be directly equated with increased available energy or energy deposition in the body (21). Because N balance and body protein deposition were not measured in this study, the specific reason why improved digestibility did not translate into improved growth performance remains unclear. Therefore, the observed improvements in ATTD should be interpreted as changes in apparent nutrient utilization rather than direct improvements in productive efficiency, and their practical nutritional significance remains uncertain.

The greater ATTD of N and GE observed at an Arg:Lys ratio of 1.10 during the early growing phase, together with the lack of further improvement at 1.20, suggests that the relationship between dietary Arg:Lys ratio and nutrient digestibility may not be simply linear. However, only three dietary ratios were evaluated in this study; therefore, the exact dose–response breakpoint cannot be determined, and an Arg:Lys ratio of 1.10 should not be defined as the optimal ratio for growing–finishing pigs. In addition, to maintain similar calculated crude protein, metabolizable energy, and lysine levels, the inclusion levels of corn, soybean meal, and some crystalline amino acids were adjusted among diets. Thus, the present results reflect the combined effects of complete diets formulated to different target Arg:Lys ratios and cannot be attributed solely to changes in arginine level. Differences in ingredient composition and crystalline amino acid inclusion may also have contributed to the observed changes in ATTD of N.

Regarding fecal characteristics, dietary Arg:Lys ratio did not significantly affect fecal score throughout the 15-week experimental period. Fecal score and fecal moisture mainly reflect fecal consistency, intestinal water status, and apparent diarrhea conditions (22, 23). Therefore, the absence of significant changes in fecal score may indicate that the dietary treatments did not cause obvious adverse effects on fecal condition. Fecal moisture was not affected at weeks 2, 5, 9, or 12, but showed a quadratic response at week 15. However, because this response was not accompanied by changes in fecal score, its biological relevance appears limited. This interpretation is supported by previous findings showing that amino acid supplementation or adjustment of amino acid balance does not necessarily affect fecal score or fecal moisture when animals are not exposed to obvious intestinal stress (24). In contrast, other studies have reported that amino acid supplementation may help improve intestinal barrier function and fecal condition under conditions of weaning stress, intestinal inflammation, or a high risk of diarrhea (25). Thus, the generally stable fecal scores observed in the present study may have limited the detection of treatment effects.

Creatine is an important N-containing metabolite related to arginine metabolism and is involved in muscle energy metabolism (26). In the present study, serum creatine concentration was not affected by dietary Arg:Lys ratio at weeks 2, 5, and 15, indicating that increasing the relative supply of arginine did not markedly alter circulating creatine levels. Similarly, some studies have found that when the basal diet meets the arginine requirement of animals, additional arginine supplementation does not significantly alter blood or tissue indicators related to creatine metabolism (27). However, other studies have suggested that under conditions of rapid growth, stress, or insufficient arginine supply, arginine, as a precursor for creatine synthesis, may influence creatine-related metabolic pathways (28). Therefore, under the present dietary and physiological conditions, different Arg:Lys ratios had limited effects on serum creatine status.

Serum homocysteine concentration at week 5 showed a significant quadratic response, with a greater value in TRT1 than in CON and an intermediate value in TRT2. This result suggests that a moderate increase in dietary Arg:Lys ratio may affect homocysteine-related metabolism (29). Previous studies have shown that homocysteine concentration can be affected by amino acid balance, methyl donor supply, and one-carbon metabolic status; therefore, changes in homocysteine concentration may reflect alterations in the regulation of amino acid metabolism (30). However, studies on the effects of dietary Arg:Lys ratio on serum homocysteine concentration in pigs remain limited, and the results under different experimental conditions are not fully consistent. Nevertheless, because the same response was not observed at week 2 or week 15, and serum creatine concentration was not significantly changed, the physiological significance of this finding requires further investigation.

## Conclusion

Overall, increasing the dietary Arg:Lys ratio from 1.00 to 1.10 or 1.20 did not affect growth performance in growing–finishing pigs. The diet with an Arg:Lys ratio of 1.10 increased the ATTD of N and GE during the early growing phase, whereas increasing the ratio to 1.20 did not further improve nutrient digestibility. However, because these responses were not consistently maintained and were not accompanied by improved growth performance, their practical nutritional significance requires further investigation.

## Data Availability

The raw data supporting the conclusions of this article will be made available by the authors, without undue reservation.
